# Supplementary figures and images for: Calcium modeling of spine apparatus-containing human dendritic spines demonstrates an “all-or-nothing” communication switch between the spine head and dendrite
Source: PLoS Comput Biol. 2022 Apr 25;18(4):e1010069. doi: 10.1371/journal.pcbi.1010069 (PMC9071165; doi:10.1371/journal.pcbi.1010069)

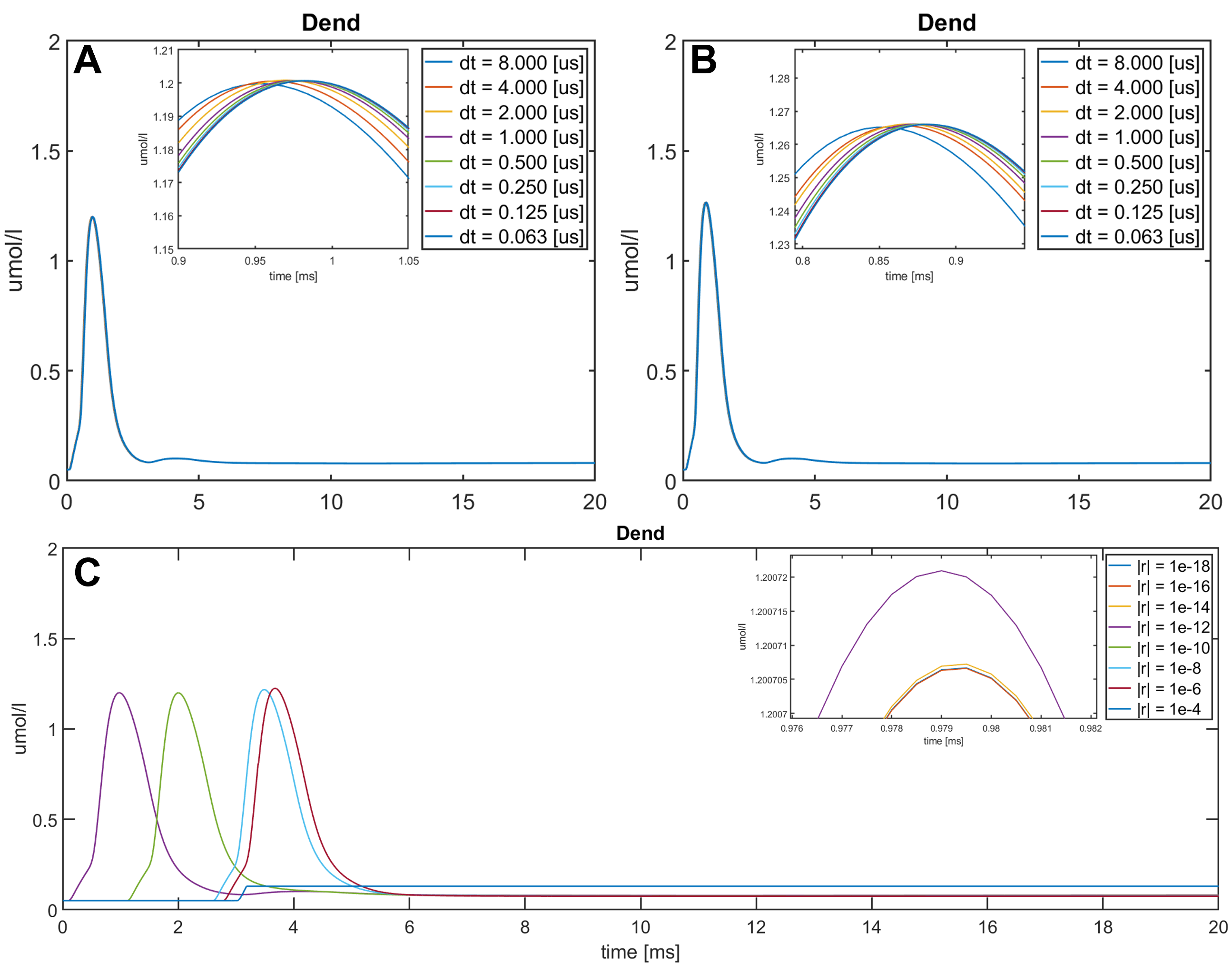

Supplement: S1 Fig — (A) Convergence of the numerical solution using the non-refined geometry, (B) convergence of the numerical solution using a refined geometry, (C) convergence using progressively smaller residual error tolerance. For (A) and (B) we decreased Δt, the maximum time step size, by powers of 2. We determined convergence when the difference in numerical solutions were near or within machine precision. (TIF) [file pcbi.1010069.s003.tif]

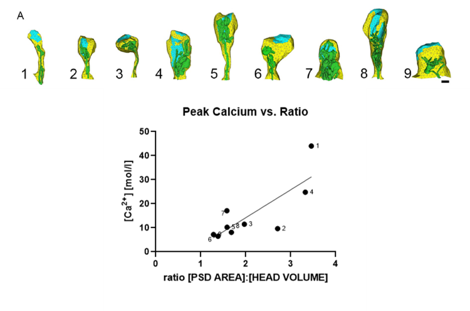

Supplement: S2 Fig — Peak calcium concentrations increase linearly with the ratio of PSD area to spine head volume. A clear outlier is spine 1 which produced above average peak calcium amplitudes in the spine head compared to all other spines. This is due to the fixed calcium influx with a particularly large PSD area, but small head volume. (TIF) [file pcbi.1010069.s004.tif]

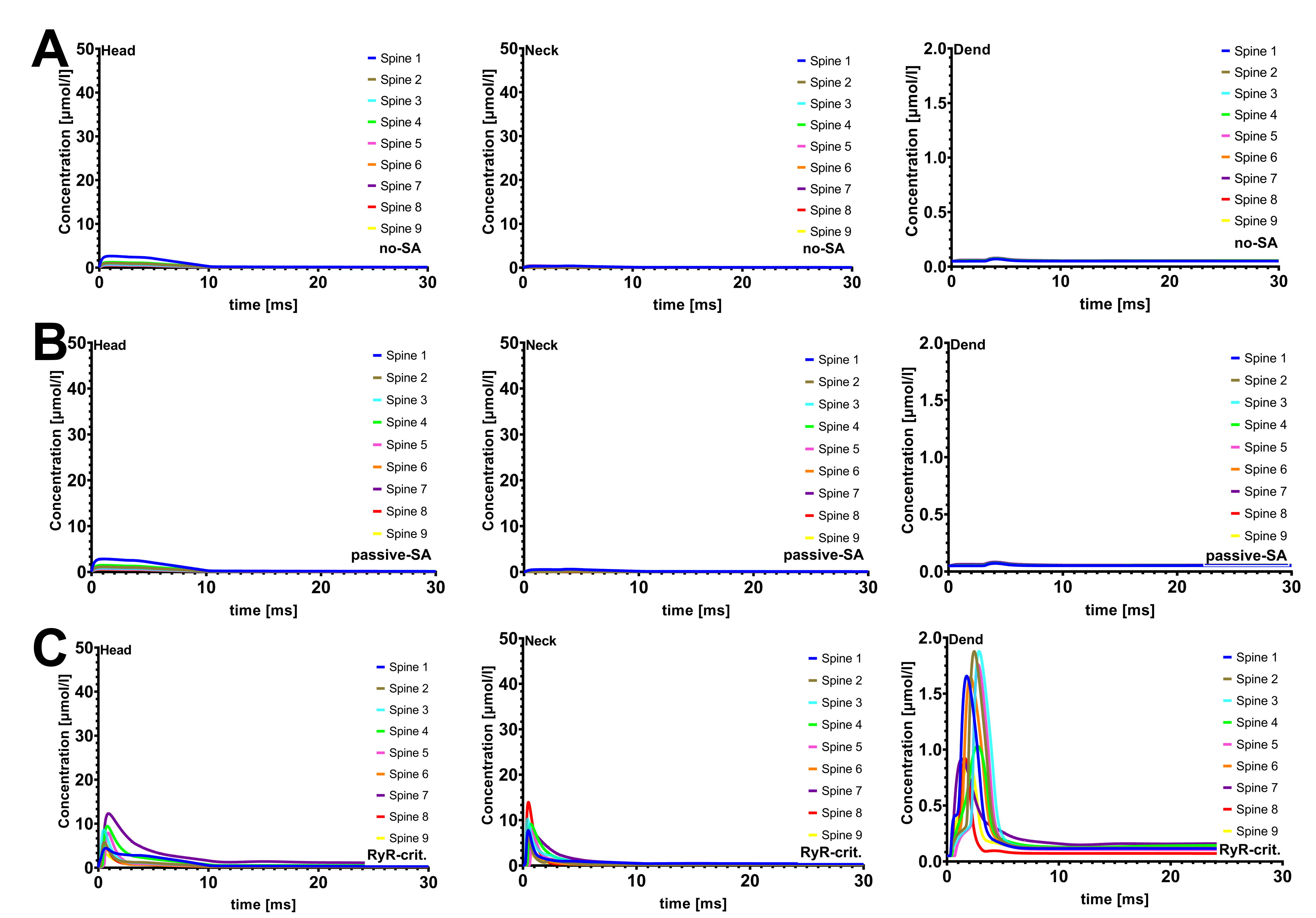

Supplement: S3 Fig — We studied the calcium responses in head, neck, and dendrite of all spines under calibrated calcium entry profiles in order to confirm that calcium profiles previously reported in [41] support our findings. For this we calibrated spine 1 to produce previously reported peak amplitudes between 0 − 14 μM in the head and used this setting on all spines. (A) Calcium dynamics in the spine head, neck, and dendrite, when no ER is present in the spine, (B) when a passive ER is added, and (C) when an active ER at critical RyR density is added. The results confirm the reported spine to dendrite coupling dynamics. (TIF) [file pcbi.1010069.s005.tif]
