# Supplementary material for: Calcium modeling of spine apparatus-containing human dendritic spines demonstrates an “all-or-nothing” communication switch between the spine head and dendrite
Source: PLoS Comput Biol. 2022 Apr 25;18(4):e1010069. doi: 10.1371/journal.pcbi.1010069 (PMC9071165; doi:10.1371/journal.pcbi.1010069)
Supplement: Attachment — Submitted filename: Response_to_reviewers.pdf [file pcbi.1010069.s006.pdf]

TEMPLE UNIVERSITY
College of Science and Technology

Department of Mathematics
Wachman Hall, Room 514
1805 North Broad Street
Philadelphia, PA 19122-6094

phone 215-204-2280
email gillian.queisser@temple.edu
web math.temple.edu/~queisser

September 20, 2021

Resubmission of "Calcium modeling of spine apparatus-containing human dendritic spines demonstrates "all-or-nothing" communication switch between the spine head and dendrite" by J. Rosado et al.

Dear Editors of PLOS Computational Biology,

Please find attached a revised version of our manuscript entitled "Calcium modeling of spine apparatus-containing human dendritic spines demonstrates an "all-or-nothing" communication switch between the spine head and dendrite", which we are resubmitting to PLOS Computational Biology.

We have addressed all comments and suggestion by the reviewers, by carrying out additional simulations, by carefully revising and extending our manuscript, adding new supplemental figures and a numerical convergence analysis, as well as a detailed point-by-point response.

We would like to thank you and the two reviewers for helping us to improve the clarity and quality of our work. We hope that our revised manuscript is now acceptable for publication in PLOS Computational Biology very much look forward to hearing from you.

With best regards,

Andreas Vlachos and Gillian Queisser

REVIEWER #1

1. In the current study, the authors use EM-based reconstructions of 9 human dendritic spines containing spine apparatus organelles to model calcium dynamics in dendritic spine. The anatomical study of reconstructed human dendritic spines revealed that the size of the postsynaptic density correlates with spine head volume and that the spine apparatus volume increases alongside the spine volume. Next, they linked these findings to spine-to-dendrite calcium communication. They showed that the calcium-induced calcium release from this intracellular organelle allows for finely tuned "all-or nothing" spine-to-dendrite calcium coupling and this process is controlled by spine morphology, neck plasticity, and ryanodine receptors. Being a neuroscientist, I am not able to assess the accuracy of the modeling part of the study. However, the motivation of the study and biological material used rise several questions which should be addressed prior to the publication:

   Response: Thank you very much for raising this important issue. The major scope of the present study has not been to systematically assess human vs. murine neocortical dendritic spines. This has been previously done by others (e.g., Benavides-Piccione et al., 2002, who show that human spines are larger and longer at higher density; or Luengo-Sanchez et al., 2018, who used modeling approaches to cluster human dendritic spines). Please note, however, that previous studies did not capture the distribution of spine apparatus organelles and specifically the 3D architecture of intracellular SA-membranes. To the best of our knowledge our study is the first to generate (laborious) 3D-TEM reconstructions of spine apparatus containing human dendritic spines that were used for Ca2+ simulations.

2. Why do authors focus on human dendritic spines?

   Response: We fully agree with the reviewer, that the choice of experimental model must be carefully considered and justified. This is especially true when a specific animal model is used to study mechanisms that may also be relevant to the human brain (in health and disease). Apparently, the best model for studying the human cortex is the human cortex, and hence we decided to assess human dendritic spines.

   Since it is currently extremely difficult (if not impossible) to carry out the required experiments in human cortical slices, a computational approach was used, based on 3D-TEM reconstructions. This is explicitly mentioned in the revised version of our manuscript. We now cite the references mentioned above–also including limitations of these studies with respect to the major scope of our study, which focused on simulations of Ca2+ signals in spine apparatus containing human dendritic spines.

   L. 269-278: "In adult human neurons, it is currently not possible to carry out the required experiments to study spine Ca2+ dynamics as they require genetic tools for simultaneous visualization of dendritic spines, SAs and changes in intracellular Ca2+ levels. *Available data from the human neocortex are mainly based on reconstructions of 2D-projected neurons (e.g., Golgi-stained or intracellularly injected neurons; Benavides-Piccione et al., 2002; Luengo-Sanchez et al., 2018) and do not capture the complex 3D architecture of SA/ER inside individual spines.* Also, it is impossible to systematically assess the relevance of individual spine and SA/ER

parameters, as they are not easy to manipulate in biologically complex systems. In this respect, computer simulations based on serial EM reconstructions provide suitable tools to explore spine Ca2+ dynamics in human dendritic spines."

It may be important to also mention in this context that our motivation is based on a recent study in which we showed that structural synaptic plasticity, i.e., changes in spine apparatuses, can be induced in acute slices prepared from neurosurgical access material of the adult human cortex (Lenz et al., 2021). We are thus confident that the reconstructions used in our study are obtained from high-quality human neural tissue preparations, and the motivation to study human dendritic spines is well-warranted.

Lines 281-287 read: "In this earlier study, we showed that (i) approximately 70% of dendritic spines contain synaptopodin clusters, (ii) synaptopodin clusters and spine apparatus organelles are found in dendritic spines with large cross-sectional areas, and (iii) a plasticity-inducing stimulus promotes remodeling of synaptopodin clusters, spine apparatus organelles, and dendritic spines in human cortical neurons. These changes correlated well with changes in spontaneous excitatory postsynaptic currents recorded from individual layer II/III pyramidal neurons [23]."

3. Are there any significant differences in morphology and/or other properties between human and rodent dendritic spines that could affect the calculations?

   Response: Studies addressing this issue are now cited in the revised version of our manuscript (c.f., line 287). Once again, the scope of this study was not a systematic assessment of rodent vs. human SA-containing spines. Therefore, our simulations are not "biased" by possible differences.

4. Do the authors expect or know that calcium dynamics in rodent dendritic spines is different? These problems should be discussed.

   Response: This warrants further investigation for reasons mentioned above and in the revised version of our manuscript. While solid experimental evidence exists, which demonstrates a role for synaptopodin and the spine apparatus in spine Ca2+ dynamics (e.g., Vlachos et al., 2009; Holbro et al., 2009; Koorkotian et al., 2014), to the best of our knowledge, currently no Ca2+ imaging data exist at single spine resolution from spine apparatus containing human dendritic spines (see technical limitations discussed in our manuscript). Future work must address the question, whether differences between murine and human SA-containing spines exists. In fact, it is conceivable that such differences may exist considering previous work - now cited in the revised version of our manuscript (e.g., Benavides-Piccione et al., 2002) - showed that human spines are larger (+60%) and longer (+30%). However, it remains to be shown whether such differences hold true for SA-containing murine vs. human spines.

   We hope this reviewer accepts our decision to not discuss these issues in more detail, since this topic goes beyond the scope of the current study, and additional experiments and/or 3D EM reconstructions of several hundred (or thousands) of human and murine dendritic spines (c.f. Luengo-Sanchez et al., 2018) might be required to convincingly address possible differences between murine and human SA-containing dendritic spines.

c.f., Lines 328-334: “It is likely that increased use of new electron microscopy techniques, such as focused ion beam, block-face serial electron microscopy, and array tomography, together with computer-based automatic 3D reconstructions of human cortical tissue will allow us to advance toward a better understanding of ultrastructure-function relations of synaptopodin associated stacked smooth ER in distinct neuronal compartments, and to test the extent to which data obtained from animal models translates to the adult human brain.”

5. It remains unclear why the authors use only 9 human dendritic spines while the published data describing morphology of rodent dendritic spines is much richer (e.g., PMID: 34428203).

   Response: Thank you for this question. We fully agree with the reviewer that FIB- or blockphase-SEM (e.g., PMID: 34428203) is a suitable approach. However, we decided to use TEM to capture the intracellular distribution of SAs as accurately as possible for a small number of spines (also enabling us to selectively change these individual parameters in our simulations). As pointed out already we focused on learning more about human dendritic spines in this study. The spines used in our study were the only 3D TEM reconstructed human neocortical spines available to us.

   We are looking forward to collaborating with other labs now that our computational pipeline has been established, and hope that the reviewer will appreciate the experimental challenges posed by our approach (especially when done in immediately immersion fixed human cortical tissue - to preserve optimal structure), where no genetic or other markers can be used to label specific structures in the tissue. This means that individual spines must be reidentified in a series of slides (at times even on different grids). As pointed out, we are confident that new advances in (T)EM techniques, data analysis and accessible data repositories will allow tackling some of the current limitations in the future.

6. If the authors chose only 9 dendritic spines, how can they be sure that such spines represent “the common types” in the population, and therefore the conclusions drawn from the model are correct and can be generalized.

   Response: We went carefully through our manuscript and made several minor amendments to avoid misunderstanding: We are not claiming that our reconstructions are representative for all spine apparatus carrying dendritic spines in the human (or murine) cortex.

   Nevertheless, the morphological correlations described in our manuscript are in line with what has been previously reported in the literature for the rodent brain + the results are robust despite the comparably small number of spines reconstructed from 7 patients. It may be important to also note in this context that we have systematically changed the morphology of the reconstructed spines to address our major scientific questions. Something that is not possible in biological experiments.

7. Accordingly, the conclusions about the tight correlations between spines parameters can be wrong due to low n number. This has been recently shown for the rodent dendritic spines (PMID: 30737431).

   Response: Thank you very much for pointing to this. In fact, this provides an interesting explanation, why the reported correlation was observed despite the seemingly small number of spines. We cite this interesting study - which admittedly escaped our attention - in the revised version of our manuscript.

   Lines 290-293 now reads: "Although this analysis is currently based on only nine reconstructed spines, the ability to detect such interrelation with small numbers indicates that these morphological measures may be tightly regulated in SA-containing spines of the human cortex (c.f., Borcyk et al., 2019)."

REVIEWER #2

In this manuscript, the authors take on a very large task of reconstructing dendritic spines from human tissue samples, reconstructing spine apparatuses from these samples, and using these geometries in simulations. Because the main conclusions rely entirely on simulations and rightly so, as the authors point out that experiments cannot be conducted in these cases, it is then important to recognize that the accuracy of the results and their interpretations rely heavily on the computational schemes used. This is where it is not clear to this reviewer if this was the best approach.

1. The authors claim that the spine apparatus sets up an 'all-or-none' connection between the spine head and the dendrite. But others have shown that only 14-19% of spines have a spine apparatus in other species (Kristen Harris' work) and I'm not sure this is even known for humans. The fraction of spines surveyed that had a spine apparatus was not mentioned in the text. But from the conclusion, those spines without spine apparatuses are at a disadvantage in humans. Is this the suggestion that the authors are making?

   Response: We appreciate the reviewer bringing up these points. The reviewer is correct in noting that the number of SA-containing spines may differ depending on the brain region (and possibly also species) studied. Our own previous work showed for example, that in the DG of the mouse hippocampus 35%-40% of dendritic spines contain synaptopodin clusters, while in CA1 10-15% show synaptopodin clusters (Bas Orth et al., J Comp Neurol 2005) – which fits well with reports of Kristen Harris and others on the number of SAs in CA1 as mentioned by this referee. Notably, the present study confirms that synaptopodin colocalizes with SAs in the human neocortex.

   Regarding numbers of synaptopodin-positive spines in the human neocortex LI 281-285 of our manuscript refer to one of our recent publications (Lenz et al., 2021): "In this earlier study, we showed that (i) approximately 70% of dendritic spines contain synaptopodin clusters; (ii) synaptopodin clusters and spine apparatus organelles are found in dendritic spines with large cross-sectional areas; and (iii) a plasticity-inducing stimulus promotes remodeling of synaptopodin clusters, spine apparatus organelles, and dendritic spines in human cortical neurons. These changes correlated well with changes in spontaneous excitatory postsynaptic currents recorded from individual layer II/III pyramidal neurons [citation 23].

   Whether it is "advantageous" or "disadvantageous" for a spine to contain an SA is not trivial to say. In fact, there has been a big dispute in the field, whether Ca2+ signals are confined to the head of dendritic spines or propagated to the parent dendrite. Based on our simulations we would argue that (i) the presence of SAs, (ii) a specific geometry of the neck region and (iii) a critical amount of RyRs is relevant for spine-to-dendrite Ca2+ communication. It is therefore conceivable that spine-to-dendrite Ca2+ propagation is overlooked when SA-containing spines are not explicitly identified/studied (e.g., Vlachos et al., 2009; Korkotian et al., 2014). This holds true specifically for brain regions with low percentages of synaptopodin/SA containing dendritic spines as noted by this referee.

2. The authors use a deterministic model for calcium dynamics, but it is well known that in small volumes, the deterministic limit may not apply. Particularly so in the SA. The authors don't mention the impact of their model choice particularly since this was the focus of extensive study — https://www.frontiersin.org/articles/10.3389/fnsyn.2015.00017/full

   Response: We thank the reviewer for highlighting the deterministic modeling choice used in this study. Stochastic model studies were carried out in the past (and are now referenced in the revised manuscript) using simplified stochastic ryanodine receptor (RyR) models. The system used in this study, in which an established continuous in time 4-state kinetic model [KEIZER] was used, merits a deterministic approach. Using parameters from [KEIZER] we arrive at RyR calcium flux rates of around 3.5e-18 mol/s which leads to ~4000 calcium ions released by all RyR on the ER membrane. For the synapse release model, we used AMPA receptor dynamics resulting in ~2400 calcium ions released at the postsynaptic density. These numbers merit a continuum-based model. Carrying out thousands of Monte Carlo simulations with very high particle numbers and then averaging produces unreasonable computational cost compared to performing the averaging in the model equations first and then computing a continuum-based equation. This is not to say stochastic effects are negligible or of no scientific significance. In fact, non-homogeneously distributed RyR or IP3 receptors and low-calcium concentration events (at the level of single particle simulations) coupled to a continuum model should be considered in forthcoming research but remain outside the scope of this structure-function study. We therefore added a paragraph in lines 415-420 to the revised manuscript contrasting existing stochastic models with the approach taken here.

   Lines 415-420: "For this work we utilized a continuum-based, deterministic model with a detailed 4-state kinetic RyR model [Keizer1996], instead of a stochastic modeling approach, with simplified RyR dynamics, as described in previous work found in, e.g., [Bartol2015]. The modeling choice is motivated by the use of AMPA receptors that release approximately 2,400 calcium ions at the PSD (using a reference spine and influx rate of $\approx 10^{-17}\ \mathrm{mol} \cdot \mathrm{s}^{-2} \mu\mathrm{m}^{-2}$ and a 10 ms time constant)."

   [KEIZER]: Keizer J, Levine L. Ryanodine receptor adaptation and Ca2+(-) induced Ca2+ release-dependent Ca2+ oscillations. Biophys J. 1996;71(6):3477-3487. doi:10.1016/S0006-3495(96)79543-7

3. Similarly, RyR dynamics are known to be stochastic (see for example https://rupress.org/jgp/article/153/4/e202012685/211900/In-silico-simulations-reveal-that-RYR-distribution) and I found it quite surprising that there was no mention of this.

   Response: We thank the reviewer for pointing out a study on calcium sparks in cardiac myocytes and agree that there is evidence for calcium signals at different spatial scales. Existing literature highlights the existence of calcium sparks [2020], calcium puffs [1999], and calcium waves [2012]. While sparks and puffs appear to be controlled by single, potentially stochastic, channel activity, calcium puffs tend to occur in channel clusters. In this study we focus on the macroscopic effect of calcium wave initiation along the endoplasmic membrane. We agree that we should more carefully distinguish these scenarios and we added a new paragraph contrasting different types of calcium events in lines 446-451.

   Lines 446-451: "The focus of this study is to determine the conditions under which stable calcium waves [Ross2012] can propagate from the spine head into the dendritic shaft. This stands in contrast to the strongly localized calcium events, such as calcium blips, sparks, and puffs, that have been studied in prior work [Veron2020, Swillens1999]. Ryanodine receptors [Keizer1996] thus are assumed homogeneously distributed across the ER membrane, leading to roughly 4,000 calcium ions released through RyR during a successful calcium wave event."

   [2020] Veron, G., Maltsev, A., Stern, M. D., & Maltsev, V. A. (2020). Elementary Intracellular Calcium Signals are Initiated by a Phase Transition of Calcium Release Channels in a Metastable State. In Biophysical Journal (Vol. 118, Issue 3, p. 256a). Elsevier BV. https://doi.org/10.1016/j.bpj.2019.11.1491

   [1999] Swillens, S., Dupont, G., Combettes, L., & Champeil, P. (1999). From calcium blips to calcium puffs: theoretical analysis of the requirements for interchannel communication. Proceedings of the National Academy of Sciences of the United States of America, 96(24), 13750–13755. https://doi.org/10.1073/pnas.96.24.13750

   [2012] Ross, W. N. (2012). Understanding calcium waves and sparks in central neurons. In Nature Reviews Neuroscience (Vol. 13, Issue 3, pp. 157–168). Springer Science and Business Media LLC. https://doi.org/10.1038/nrn3168

4. Calibration with known calcium dynamics from the literature — this to me was the strangest part of the model — the authors show peaks of up to 50 \mu M!! The model was not calibrated to known calcium transients in the literature nor were any clarifications provided about how the different parameters would affect the outcome.

Response: In this study we collected data and parameters from various sources (e.g., Luzzi1998, Muller2005, Chiu1980, Graupner2003, Fink2000, Keizer…) to calibrate most model components as closely to previous research as possible. The parameters we did vary in this study were RyR density, maximum calcium influx, and buffering capacity. We intentionally did not tune the other model parameters to fit experimental transients observed under different conditions, since we were interested in studying how different morphological configurations may affect calcium transients and peak amplitudes. For instance, the spine reconstructions from human dendritic spines are noticeably different from those shown in [Bartol]. In [Bartol] spine ER consisted of protrusions of smooth ER into the spine while reconstructions in this study showed large and restrictive SA occupancy in the spine neck and head, which was something we wanted to study in terms of calcium signaling. Another important factor is the size of the postsynaptic density (PSD). Based on the reviewer's comment we plotted peak calcium with respect to the PSD area/spine head volume ratio, which shows that peak calcium correlates with this ratio. The spine with the highest peak amplitude is a clear outlier. In the revised manuscript we more carefully discuss this point in lines 150-162.

Another important point is the calcium influx dependence on PSD size, which is discussed in Figure 4. Figure 4 calcium transients were computed using a synaptic calcium influx model that was PSD size-independent, in contrast to Figure 3 (PSD-dependent dynamics). The data shows peak amplitudes roughly 5-fold lower, and close to previously observed amplitudes (e.g., in [Bartol]). We admit that this conclusion was not well described, and we therefore modified manuscript to present the difference clearly between PSD-dependent vs. PSD-independent calcium influx to the reader (lines 150-162).

Overall, the interesting observation of this study is that the morphology of spines, the ER, the size of the PSD can produce diffusion-restricted and PSD-size controlled calcium profiles that are tuned by cellular architecture.

Lines 150-162: "For our studies we collected parameters from [Luzzi1998, Muller2005, Chiu1980, Graupner2003, Fink2000, Keizer1996, Breit2018, Keller2008] and calibrated model components to previous research. Parameters varied in this study were the RyR density, maximum calcium influx, and buffering capacity, to dissect how different morphological configurations may affect calcium transients and peak calcium amplitudes. It should be noted that one of our spines (spine 1) produced a maximum calcium amplitude in the range of 50 microM which stands in contrast to previously observed values reported in [Bartol2015]. This discrepancy can be explained by the fact that a constant calcium influx was used for each spine (Figure 3), so that spine 1, with an exceptionally large PSD (PSD size scales the total number of calcium ions released) together with a small spine head volume, presents a strong outlier (see Supplemental Figure S4). When adjusting calcium influx by PSD size the maximum calcium amplitude of all spines is reduced up to 10-fold (see Figure 4), which confirms previously reported ranges."

New figure (for question #4)

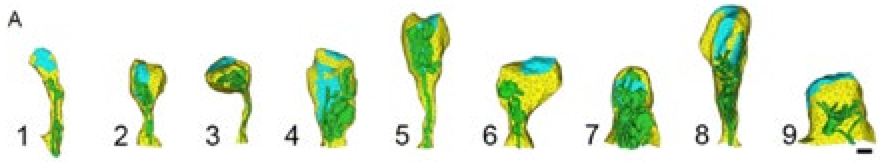

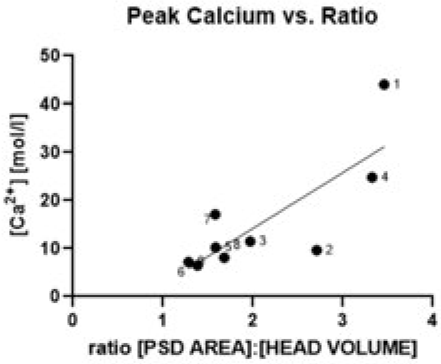

[Bartol]: Bartol, Thomas M et al. "Computational reconstitution of spine calcium transients from individual proteins." Frontiers in synaptic neuroscience vol. 7 17. 7 Oct. 2015, doi:10.3389/fnsyn.2015.00017

5. Mesh generation: How do the mesh generation methods preserve the original geometry? This is not discussed. Generating surface meshes is not as easy as the authors make it out to be — see https://journals.plos.org/ploscompbiol/article?id=10.1371/journal.pcbi.1007756 For example.

Response: We fully agree with the reviewer that 3D reconstruction from microscopy data and the construction of surface and volume meshes is a nontrivial task. The pipeline developed in this paper generates a contour set from the raw microscopy stack using ImageJ. The contours are imported into ProMesh to generate surface triangulations and tetrahedral volume grids. Two main objectives were followed: 1. Retaining the original geometry and 2. Generating volume elements that are maximally isotropic to ensure good numerical convergence properties. From a raw surface mesh, we carry out a remeshing step with an edge length suitable for preserving the detailed 3D morphologies of spine and spine apparatus. The volumetric meshes are constructed under an aspect ratio constraint and smoothing step similar to what is presented in [Lee]. We added a paragraph in lines 382-401 dedicated to the reconstruction pipeline that describes all steps involved in the construction of high-quality computational meshes.

Lines 382-401: "To ensure that surface and volume discretizations are suitable for numerical simulations the following procedural steps were followed to process the raw meshes, which consist of the subsets spine, dendrite, synapse, and ER. All steps are carried out using [Reiter2012]:

[1.] Dendrite surface remeshing with edge lengths in the range 15-30 nm, and the spine, synapse, and ER re-meshed with edge lengths in the range 5-10 nm.

[2.] Some parts of the ER may intersect the plasma membrane (from the semi-automatic procedure used above with [Schindelin2012, Cardona2012] and is corrected by manually moving the vertices/edges of the ER.

[3.] Consistency checks: remove potential double edges, double vertices, stray vertices, and close holes in the geometry.

[4.] Remove very small edges (this may arise when remeshing occurs) and mesh holes.

[5.] Remove sharp surface angles by a) removing critical vertices and remeshing or b) perform one iteration of Laplacian smoothing.

[6.] Tetrahedral mesh generation with a minimum dihedral angle as large as possible to avoid sliver triangles or obtuse angles.

[7.] Assign subsets to the geometry for simulation and measuring zones, i.e., cytosol, dendrite, head, etc.

For the Laplacian smoothing we perform the algorithm described in [Hansen2005], which is the structured Laplacian smoothing algorithm implemented in [Reiter2012].

[Lee] Lee, C. T., Laughlin, J. G., Angliviel de La Beaumelle, N., Amaro, R. E., McCammon, J. A., Ramamoorthi, R., Holst, M., & Rangamani, P. (2020). 3D mesh processing using GAMer 2 to enable reaction-diffusion simulations in realistic cellular geometries. In H. Berry (Ed.), PLOS Computational Biology (Vol. 16, Issue 4, p. e1007756). Public Library of Science (PLoS). https://doi.org/10.1371/journal.pcbi.1007756

6. Numerical convergence — how do the authors know that the simulations converged despite all the geometric complexity and non-linearity of the equations?

Response: The presented numerical results were obtained by using a Bi-CGSTAB [VanderVorst] Krylov-type linear solver with Gauss-Seidel preconditioning together with a standard backward Euler time-stepping scheme. The Bi-CGSTAB iterative solve was set to a maximum of 10,000 iterations and was used to solve the nonlinear implicit problem at every time step. We used the 2-norm of the residual r in our convergence checks and set the tolerance to 10^{-18}. To test for numerical convergence, we carried out a sequence of test simulations with decreasing time step size and increased grid resolution (decreased edge length). The time convergence sequence we did is now presented in a new supplemental figure in the revised manuscript. Based on these convergence studies a suitable time step of 0.5 µs and a grid resolution was chosen, such that the next refinement level produced at most 5% difference in the peak calcium concentration and the identified critical RyR densities remained unchanged. Typical geometries contained on the order of 1,000,000 grid vertices. We added more details about the numerical setup and the convergence analysis in lines 493-514, including a new supplemental figure, to the revised manuscript.

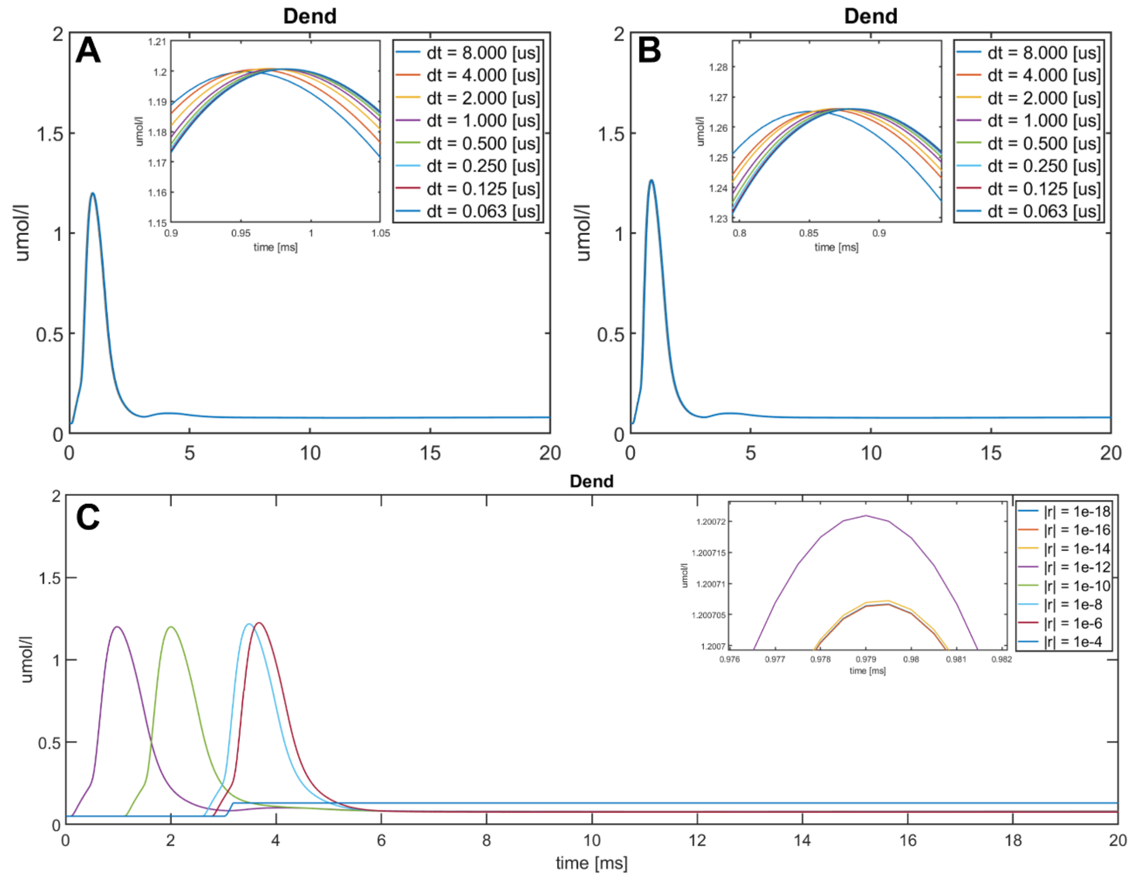

Lines 493-514: "For the presented results, the linearized problems were solved using a Bi-CGSTAB [vanderVorst1992] solver using Gauss-Seidel preconditioning, while time integration was realized using the implicit backward Euler scheme. The Bi-CGSTAB iterative solver was set to a maximum of 10,000

iterations and is used to solve the implicit problem at every time step. The implicit problem can be written as an implicit update rule

$$c_c(t) = c_c(t - \tau) + \tau F(c_c, t)$$

where ‘t’ is the next time step, ‘$t - \tau$’ is the current time step, and F() is the right hand side of the system of equations. For the time integration we used adaptive time-stepping with a prescribed simulation time step size τ_{sim} , where a change in the time step size is governed by performing a convergence check for the implicit solve of the nonlinear system of equations. For each simulation the minimum time step size is set by $\tau_{min} = \tau_{sim}/2^{15}$. The measure of the residual norm was used to determine the value of the convergence check. The implicit equation is of the form

$$c_c(t - \tau) + \tau F(c_c, t) = 0 \Rightarrow Ax - b = 0$$

where $r_n = Ax_n - b$ is the residual [Trefethen1997], x_n is the approximation after ‘n’ iterations of Bi-CGSTAB, using a tolerance of $\epsilon = 10^{-18}$. We performed convergence analysis by a sequence of test simulations in which the time step size τ was halved (Figure S3). In supplemental Figure S3 we show the convergence of the solutions by decreasing the time step size by powers of 2 (see Figures S3-A, S3-B) and decreasing the error tolerance (Figure S3-C). In Figure S3-C we choose the smallest tolerance such that the difference in solutions were within machine precision. Based on the convergence results we chose a standard time step size of τ=0.5 microseconds since differences in the calcium concentrations between this value and the next were less than 1/100-th micro mols and there was no discernible difference with our determined critical ryanodine receptor values.”
