# Supplementary material for: Calcium modeling of spine apparatus-containing human dendritic spines demonstrates an “all-or-nothing” communication switch between the spine head and dendrite
Source: PLoS Comput Biol. 2022 Apr 25;18(4):e1010069. doi: 10.1371/journal.pcbi.1010069 (PMC9071165; doi:10.1371/journal.pcbi.1010069)
Supplement: Attachment — Submitted filename: Response_to_reviewers.pdf [file pcbi.1010069.s007.pdf]

EDITOR

In light of the reviews (below this email), we would like to invite the resubmission of a significantly revised version that takes into account the reviewers' comments. The authors need to address all the reviewer 2 comments:

Response: Thank you for the opportunity to clarify the additional points and re-submit a revised version of our manuscript. New simulations were run too and additional amendments were made to the text. Please find below a point-to-point response.

1. Incorporate in the manuscript the authors' response to comment 2 from the first revision (the reader must be sure, why the deterministic approach to simulating calcium in the spine is not correct).

   Response: Done! We addressed this point and provided a response to Reviewer #2.

2. Address comment 4 with additional simulations showing that the model is well calibrated and explain 50 uM Ca in the spine in Fig. 5.

   Response: We ran additional simulations to address this point. Simulation details are provided in the response to Reviewer #2. The results are compiled into a new supplemental figure 5 (which is also found at the end of this document), along with new text explaining the findings. All findings support the original data presented in Fig. 3 and Fig. 4 and substantially strengthens the results of this paper.

3. Possibly add some stochastic simulations to further assure the reader that stochastic effects do not play a huge role in their simulations.

   Response: We agree that the differentiation between stochastic and deterministic approaches deserves attention. We therefore added a new paragraph to the manuscript which reports the calcium ion numbers involved in calcium wave initiation and contrasts these effects with small scale effects, like calcium sparks and puffs. Our analysis is further supported by Reviewer #3 who writes "*The questions concerning the possible use of stochastic models are well justified and reasonably answered by the authors from my point of view.*" We acknowledge that Reviewer 2 provided references reporting results of stochastic simulations. These studies involve RyR dynamics which have in the literature been reported to produce calcium sparks, puffs, which may be controlled by single or small clusters of RyR and merit stochastic modeling (we cite these papers in our revised manuscript). In our studies we examine the macroscopic behavior of calcium waves throughout larger structures such as the spine, head, neck, dendritic shaft, ER membrane, which can be described at the continuum level. The differentiation between RyR activity at different scales is now part of the revised manuscript.

Added paragraph (lines 425-442): *"Using the parameters from [36] we arrive at RyR calcium flux rates of around 3.5 ×10−18 mol/s which leads to ≈ 4000 calcium ions released by all RyRs on the ER membrane. Additionally, previous literature highlights the existence of calcium sparks, puffs, and calcium waves; while sparks and puffs are controlled by, potentially stochastic, clustered channel activity our studies focus on the macroscopic effects of calcium wave initiation along the endoplasmic membrane which constitutes a significantly higher number of calcium ions released into the cytosol. [...] These numbers merit a continuum-based model. Carrying out thousands of Monte Carlo simulations with very high particle numbers and then averaging produces unreasonable computational cost compared to performing the averaging in the model equations first and then computing a continuum-based equation. This is not to say stochastic effects are negligible or of no scientific significance. In fact, non-homogeneously distributed RyR or IP3 receptors and low-calcium concentration events (at the level of single particle simulations) coupled to a continuum model should be considered in forthcoming research but remain outside the scope of this structure-function study."*

REVIEWER #1

The authors addressed all my questions in a satisfactory way.

Response: We thank the reviewer for providing comments and questions regarding the biological components and explanations of the manuscript. We are happy that the reviewer's questions and comments were satisfactorily addressed.

REVIEWER #2

I am honestly not sure what to make of the revised manuscript. In the response to reviewers, the authors agree that all the concerns I raised are valid and important. But the revised manuscript has minimal text changes but a very minimal attempt to ponder if considerations of the concerns raised could alter the results and predictions. I'll provide a specific example — the authors state that they choose not to calibrate parameters to ensure that the calcium concentrations were constrained to prior observations. "We intentionally did not tune the other model parameters to fit experimental transients observed under different conditions, since we were interested in studying how different morphological configurations may affect calcium transients and peak amplitudes." This is worrisome because the strength of the conclusions and predictions rely on such calibrations. How can the role of morphological configurations be assessed if the model is not calibrated?
In my assessment, it appears that in the private response to reviewers, the authors agree that all the points raised, in fact, by both reviewers are quite important. But in the public document, which is the manuscript, the concerns appear to be swatted away.

Response: We thank the reviewer for the suggestions and clarifications, helping us to further improve the clarity and quality of our work. In the revised version of the manuscript, we addressed the remaining points by following the editors' request to

a) Incorporate in the manuscript the authors' response to comment 2 from the first revision (the reader must be sure, why the deterministic approach to simulating calcium in the spine is not correct).

Response: In order to better discuss the choice of a deterministic approach in the manuscript itself we followed the advice by the editor and now added the response of our previous revision as new text in the revised manuscript. The new paragraph can be found in lines 425-442.

Added text (lines 425 – 442): *"Using the parameters from [36] we arrive at RyR calcium flux rates of around 3.5 ×10−18 mol/s which leads to ≈ 4000 calcium ions released by all RyRs on the ER membrane. Additionally, previous literature highlights the existence of calcium sparks, puffs, and calcium waves; while sparks and puffs are controlled by, potentially stochastic, clustered channel activity our studies focus on the macroscopic effects of calcium wave initiation along the endoplasmic membrane which constitutes a significantly higher number of calcium ions released into the cytosol. [...] These numbers merit a continuum-based model. Carrying out thousands of Monte Carlo simulations with very high particle numbers and then averaging produces unreasonable computational cost compared to performing the averaging in the model equations first and then computing a continuum-based equation. This is not to say stochastic effects are negligible or of no scientific significance. In fact, non-homogeneously distributed RyR or IP3 receptors and low-calcium concentration events (at the level of single particle simulations) coupled to a continuum model should be considered in forthcoming research but remain outside the scope of this structure-function study."*

b) Address comment 4 with additional simulations showing that the model is well calibrated and explain 50 uM Ca in the spine in Fig. 5.

Response: We appreciate the comments raised by the reviewer and we agree with the concerns that were raised. While it remains central to this study that we use previously verified calcium entry dynamics, the reviewer raises an important point, i.e., to verify that under calibrated calcium entry spine to dendrite coupling can still be observed. We therefore ran additional simulations whereby the calcium influx of outlier spine 1 was calibrated to values reported in *Computational reconstitution of spine calcium transients from individual proteins* by Bartol, Keller, Kinney, Bajaj, Harris, Sejnowski, and Kennedy. This calcium profile was then applied to all spines (all of which show peak amplitudes in the spine head that match previously reported ranges). We then ran simulations under the assumption of a) no ER, b) passive ER, and c) active ER. The results of these simulations are now reported in a new supplemental figure 5 (also provided at the end of this document). As can be seen from the new figure, all spines still behave as reported in the main manuscript (see Fig. 3 and 4). We further added a new paragraph describing the findings under calibrated calcium entry in lines 161 – 168.

Added text (lines 161 – 168): *"Given that the Ca2+ concentration in the head scales with the total number of ions released into the spine head, the density of AMPA receptors, and the size*

and shape of the spine head, we were interested in spine responses when providing a uniformly calibrated Ca2+ signal. We therefore calibrated Ca2+ influx for outlier spine 1, such that the peak Ca2+ amplitude lies within ranges reported in [42] and used this influx for all spines. As shown in Supplemental Figure S5, the peak amplitudes in the head all lie within the reported range, and the Ca2+ dynamics in neck and dendrite confirm the spine to dendrite coupling effects shown in Fig 3 and Fig 4."

c.f., new figure: supplemental figure 5

REVIEWER #3

The revised version extends the papers in terms of properties of the model and its application. As a numerical reviewer, I find the added discussion points very helpful to better understand the model choices made, but I cannot comment on the biological aspects.

The questions concerning the possible use of stochastic models are well justified and reasonably answered by the authors from my point of view.

I also appreciate the added section on the numerical aspects of the solution process. I suggest nevertheless to clarify some details (see below) in a minor revision.

Response: Thank you for the careful and critical evaluation of our manuscript and the commendation of our work. We addressed each of the additional remarks below.

1. The measure of the residual norm was used to determine the value of the convergence check. Please reformulate. Maybe: "The Euclidean norm of the residual was used to check for convergence"?

   Response: We have reformulated this statement and can be found at 576 of the revised manuscript:
   The Euclidean norm of the residual was used to check for convergence.

2. 10e-18 is a quite strict tolerance. It is "nice to have", but is it necessary? Is this high accuracy for the linearized problems required in order to get convergence of the resulting trajectories?

   Response: In supplemental figure S3 a convergence plot using varying tolerances, subfigure C, is now provided. We ran sample simulations using a larger tolerance ~ 1e-12 and the results remained unchanged; however, we observed no measurable additional computational cost (i.e. runtime) when using the smaller tolerance 1e-18. Therefore, out of caution we decided to use stricter tolerance.

3. "The implicit equation is of the form c_c(t-\tau)+\tau F(c_c(t),t) = 0 => Ax-b =0 where r n= Ax n − b is the residual" Please clarify the relation between A and F. I assume that A is the linearized Model (Jacobi matrix)?

Response: We elaborated on the equations shown after line 576 and explained that the matrix is the corresponding linearized problem using a Jacobi matrix. The added text for points 3 and 4 begins on line 583 in the revised version of our manuscript.

Added text (lines 583 – 587): *"This tolerance was chosen to ensure accuracy of the linearized problem and to ensure convergence of the trajectories. In the equation above the matrix M is the matrix corresponding to the linearized problem of $F(\cdot)$ using a Jacobi matrix. When we collect all the terms to one side of the equality we obtain an equation of the form $Ax - b = 0$."*

4. A Newton iteration is applied in each time step with bi-cgstab as inner solver? Or is only one linear problem solved per time step?

   Response: Correct! A Newton iteration is applied in each time step with Bi-cgstab as the inner solver. The sentence at line 569 was expanded upon.
   Once again, we would like to thank the editor and the three reviewers for their careful and critical evaluation of our manuscript and for their commendation of our work. Indeed, this helped us to significantly improve the clarity and quality of our work. Hoping that our revised manuscript is now suitable for publication we are looking forward to hearing from you.

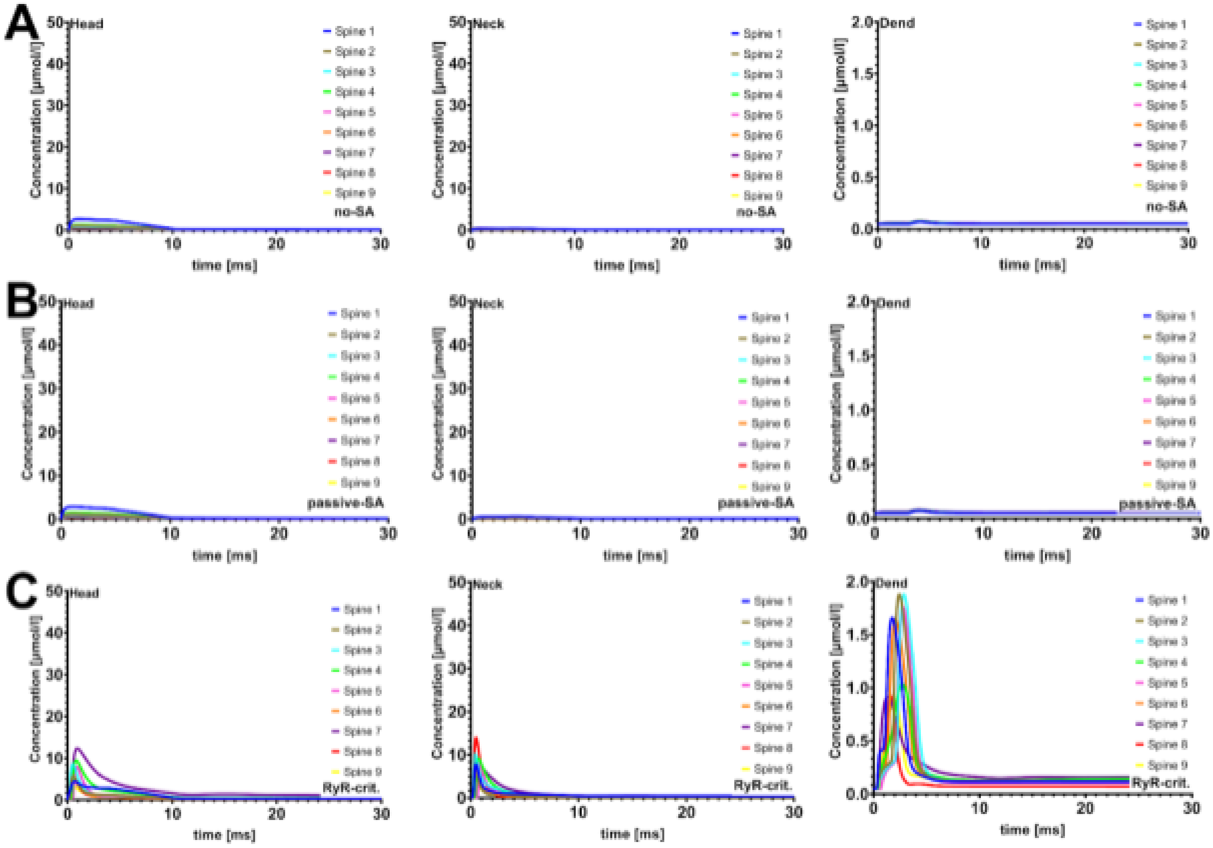

Supplementary Figure 5
